# Getting a Grip on NCDs in China: an Evaluation of the Implementation of the Dutch-China Cardiovascular Prevention Program

**DOI:** 10.1007/s12529-014-9453-z

**Published:** 2014-12-04

**Authors:** Xuefeng Zhong, Bert Potemans, Lianzhi Zhang, Brian Oldenburg

**Affiliations:** 1 Anhui Provincial Center for Disease Control and Prevention, Anhui, China; 2 The Hague, The Netherlands; 3 School of Public Health and Preventive Medicine, Monash University, Monash, Australia; 4 School of Population and Global Health, University of Melbourne, Melbourne, Australia; 5 China-Australia NHMRC, Canberra, Australia

**Keywords:** Community-based approach, Cardiovascular disease, Risk factors, Screening, Primary care, Intervention

## Abstract

**Purpose:**

China has experienced a rapid increase in cardiovascular diseases and related chronic conditions over the last 20 years, and there is now an urgent need for new approaches that can effectively reduce the progression of cardiovascular disease in high-risk individuals. This article reports on the evaluation of the implementation of the Dutch-China Cardiovascular Prevention Program.

**Methods:**

A screening questionnaire, based on the Dutch concept of *Prevention*-*Consultation*, was distributed among inhabitants in seven communities in Anhui Province, China. This tool categorizes individuals into being at low or high risk according to their responses to a number of self-administered questions. The “high-risk” individuals were then invited to undergo further clinical and laboratory tests, before being offered lifestyle education and counseling. The program is delivered by primary care professionals from local community health service centers (CHSCs). An evaluation of the program’s implementation was undertaken with both program participants and CHSC staff.

**Results:**

The screening questionnaire was completed by 9067 participants in seven demonstration communities. Thirty percent of these individuals were categorized as high risk according to their scores. About one third of these individuals returned for further clinical and laboratory tests. Almost half of those re-screened participated in lifestyle education classes. Program participants and community health staff provided mostly positive feedback about the program.

**Conclusions:**

While the program proved acceptable and feasible for delivery by CHSCs and by program participants, additional strategies are required to improve future uptake of both screening and subsequent lifestyle education by those at high risk.

## Introduction

Cardiovascular (CVD) and other non-communicable chronic diseases have become the leading causes of mortality and disease burden worldwide with the majority of this burden occurring in low- and middle-income countries [1]. Major community-based cardiovascular disease prevention programs have now been successfully conducted and evaluated in Finland, the USA, and many other developed countries since the early 1970s [2–4]. However, with rapidly aging populations and changing lifestyles that include increases in tobacco smoking, unhealthy diet, excessive alcohol consumption, and a reduction in physical activity, the burden of CVD and other associated chronic conditions are now also rapidly increasing in most developing countries as well [1]. Therefore, the WHO has launched a *Global Action Plan for the Prevention and Control of Non*-*communicable Diseases* to achieve a very ambitious worldwide reduction of 25 % in mortality from non-communicable diseases (NCDs) by 2025 [5].

China has experienced a rapid epidemiological transition in recent years, and chronic conditions have now also become the major causes of disease burden in that country in the last decade. The lifestyle changes leading to these major changes in disease trends are mainly due to China’s rapid socioeconomic development and urbanization, which are occurring at an unprecedented rate. This increasing burden of chronic diseases and the associated preventable premature morbidity and mortality in China will continue to have a very substantial impact on healthcare costs, the labor force, and the country’s economy over the coming years [6, 7]. To address this challenge, China’s *National Plan for NCD Prevention and Treatment* (2012–2015), issued by the Ministry of Health and 14 other ministries and state administrations in May 2012, proposes the implementation of an extensive and ambitious list of strategies in China in order to slow down and even reverse these trends over the next 10 years [8]. However, there are a number of very significant challenges in the implementation of these in China. Firstly, as already mentioned, the rapid urbanization and modernization of China is contributing very significantly to the epidemiological crisis of chronic NCDs [9]. Secondly, the majority of the adult Chinese population already has multiple risk factors for CVD and other NCDs [10]. Thirdly, while the number of people diagnosed with one or more chronic diseases is increasing rapidly in China, most are not well-controlled and many people remain largely unaware of their level of risk [8]. Finally, China’s healthcare system and public health institutes currently lack the human resources, knowledge, and capacity to prevent and control NCDs effectively [11]. Therefore, the need to identify and evaluate effective, affordable, and scalable approaches to prevent and control chronic diseases, such as CVD and diabetes, is very urgent [12].

In China’s Anhui Province between 2009 and 2013, Dutch and Chinese experts have collaborated on the development and evaluation of a new approach to prevent and control CVDs and related conditions. This approach has been extensively adapted from strategies that have been previously developed and very well evaluated from the Netherlands and other developed countries over the past 30 years [13]. However, it is not currently known how well this kind of approach might transfer to Chinese communities and with their workforce of health professionals. This paper describes the development and evaluation of the implementation of this new approach in China and discusses its future wider implementation scalability.

## Methods

### Setting and Program Context for the Dutch-China Cardiovascular Prevention Program

**Fig. 1:**
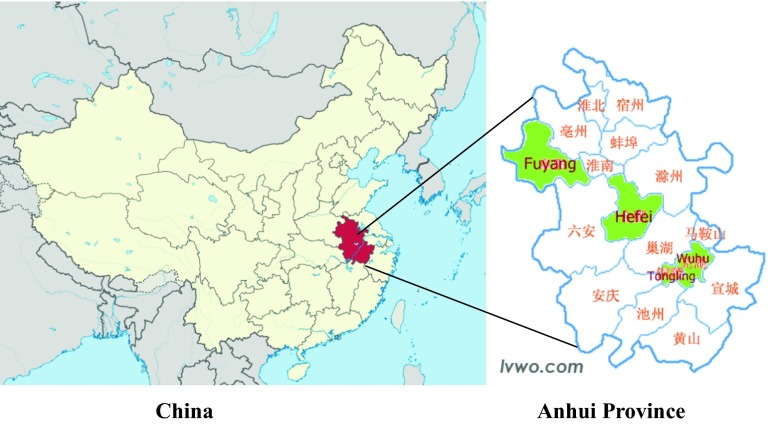
Map of China and Anhui Province

The program was undertaken in Anhui Province, China which has a population of almost 70 million people in 2010 [14]. Anhui is located in the central-eastern region of China and has 16 cities and 105 counties (Fig. 1). In terms of socioeconomic development, Anhui is “mid-way” between the rapidly developing provinces that are primarily located along the east coast of China, and Western China, which is generally less well developed. Each of these cities has six to eight districts or counties, and each district has four to five communities with approximately 20,000–30,000 residents. Each community has its own Community Health Service Center (CHSC), and each of these has four to six Community Health Service Stations (CHSSs). The latter are most responsible for the delivery of primary healthcare and health education to the residents in their local community. In China, these programs and services are generally organized according to geographic neighborhoods and housing complexes, and teams of health professionals are assigned to provide these to the residents in these communities.

China also has a nationally coordinated Center for Disease Prevention and Control (CDC) system which has four levels across the whole country: (1) the national CDC, (2) the provincial-level CDCs, (3) the city-level CDCs, and finally, (4) the district- or county-level CDCs. The latter level of the CDC system is particularly important for supporting the delivery of public health and primary care services such as those described in this manuscript. The CDC system also has the responsibility for supporting, training, and supervising the CHSCs and their associated CHSSs in the delivery of basic primary care, health education, and health promotion related to the prevention and control of chronic diseases. However, well-designed community-based programs targeting individuals who are at high risk of chronic conditions like cardiovascular disease are still in their infancy in China.

### Description of *Dutch*-*China CPP* and Its Implementation

The development and implementation of the Dutch-China Cardiovascular Prevention Program (Dutch-China CPP) was coordinated by a team from the Netherlands in partnership with the Anhui Provincial CDC, a program funded by the Dutch Government during the years 2009–2013.

The initial piloting and testing of the program and its delivery was undertaken in the Heyedi community in the Shushan district of Hefei City, which is the capital of Anhui Province. After a 24-month piloting and adaptation phase from June 2010 to June 2012, the program delivery model was further refined and then implemented in a further seven communities in three other cities (Fuyang, Tonglin, and Wuhu) in Anhui Province. The program was delivered by the health professionals from the CHSC in each of the selected communities. Individuals of at least 35 years and living in the neighborhood of the relevant CHSCs were invited to participate. The Community Neighborhood Committee, local social organizations, local employers, and institutions such as schools also supported the program and the mobilization and recruitment of participants.

### Program Piloting and Adaptation Phase

#### Self-Assessment Questionnaire

Because it was not possible to extract risk factors in an automated fashion from existing patient medical records, it was decided to pilot an alternative screening test for cardiovascular risk from July 2009 to June 2011. Using a previously validated Chinese cardiovascular risk assessment tool [15, 16] that combines measurement of seven risk factors—i.e., gender, age, smoking, systolic blood pressure, body mass index, glucose, and total cholesterol—the pilot CHSC evaluated this approach in their local community. Individuals were either recruited in public areas like markets or when they visited the outpatient clinic of the CHSC for other reasons. However, the number of participants using this approach was very small and it also required blood collection to measure cholesterol; following this, CHSC nurses then directly visited individual households in the community. Although the number of recruited participants increased using this recruitment strategy, the strategy was still very labor intensive and it was agreed that this approach would not be scalable in the future due to the human resources that would be required for its widespread implementation. Therefore, following review of the recruitment strategies used in the pilot study and the problems also associated with the measurement of cholesterol, it was agreed to adapt and evaluate the Dutch Preventive Consultation concept [13, 17], which involves a two-step risk stratification process. Firstly, a brief five-item self-report questionnaire—that is, age, BMI, smoking habits and personal and family history—is completed by respondents (*step 1*). Secondly, those individuals with a score that is indicative of high risk were invited by phone to attend an individual clinical assessment (*step 2*). Finally, for the China program, individuals whose scores were above a pre-defined cutoff score were also invited to attend group health education and lifestyle education sessions (*step 3*).

#### Risk Stratification

Steps 1 and 2 were adapted from the Prevention-Consultation approach developed by the Dutch Association of General Practitioners over the last 5 years [13, 17]. The risk tool used in the Netherlands was originally developed by combining two European tools which have been extensively evaluated and validated over more than 10 years. These two tools are (1) SCORE [18], a tool that calculates the risk for cardiovascular diseases in the next 10 years based on age, gender, smoking, blood pressure, glucose, and cholesterol and (2) FINDRISK [19], a tool that calculates the risk of diabetes mellitus based on body mass index, waist circumference, and personal and family history. Based on extensive research conducted with the FINDRISK tool [19] in Finland and its adaptation to many other countries over the past 10 years, it has been established that a number of the most important risk factors can be assessed by a self-completed five-item questionnaire. The response to each item is given a score, and the overall score or risk estimation involves simply summing the scores for each item (Table 1). The maximum score on this tool is 10, and the threshold for further testing when the tool was adapted to the Chinese context was set at 5 or more. To achieve a score of 5 or higher, older respondents required at least one major risk factor in addition to age and younger individuals required at least a combination of other risks factors, in order to be identified as requiring further testing and education. Based on the pilot data, a cutoff of 5 was used because it was estimated that this would identify at least 20–25% of the initially screened population for further testing. Although this tool is not able to generate a precise measure of cardio-metabolic disease risk, its purpose is to identify a subgroup of participants who are likely to have a moderate to high cardio-metabolic risk and who have at least one unhealthy lifestyle risk factor that can be subsequently targeted in order to reduce risk. Table 1 shows the items used in the China Self-Assessment Questionnaire (China-SAQ) and the corresponding scores that were developed for the Dutch-China CPP. Once self-completed, each individual’s questionnaire responses were then entered into a computerized database by CHSC staff and a risk score was calculated for each individual.

**Table 1:** Scoring of China CVD risk self-assessment questionnaire (adapted from SCORE and FINDRISK tools)

Items	Level assortment	Score
Age	<35 years	0 point
	35–44 years	1 point
	45–54 years	2 points
	≥55 years	3 points
BMI	<24	0 point
	≥24 and <28	1 point
	≥28	2 points
Smoking	Yes	2 points
Personal history of DM and/or hypertension	Yes	2 points
Family history of DM and/or CDV diseases	Yes	1 point

#### Follow-up Consultation

Each individual with a score exceeding the set threshold score (i.e., 5 or more) was then invited to attend the next step (*step 2*—*follow*-*up consultation*). At this face-to-face consultation, the previously completed questionnaire responses were re-administered and the individuals were asked further questions about their personal and family medical history in relation to ischemic heart disease, stroke, hypertension, and diabetes. A number of additional clinical measures were also taken, including body mass index, waist circumference, blood pressure (measured twice with an interval of at least 5 min and the average being used), capillary blood glucose (fasting or at random), and cholesterol (optional). If blood pressure or blood glucose exceeded specified values (average systolic BP exceeded 140 mmHg, fasting glucose exceeded 7 mmol/ml or the non-fasting glucose exceeded 11 mmol/ml), participants were then offered an appointment for a second measurement. If participants still had high blood glucose or blood pressure, they were then referred for further clinical investigation and potential treatment. Before the end of the consultation, the complete risk profile and follow-up actions were discussed with the individual.

#### Lifestyle Education Group Sessions

All participants who had a confirmed risk score of at least 5 during the consultation session were then invited to attend lifestyle education group sessions conducted by appropriately trained health professionals from the community health centers (*step 3*—*Lifestyle education and counseling*). Table 2 summarizes the components of these sessions.

**Table 2:** Overview of lifestyle education group sessions

1. Welcome
2. What are cardiovascular diseases (stroke, ischemic heart diseases) and diabetes? Goal: to increase information and awareness. Method: short video films followed by a brief discussion. (Animation and spoken word).
3. What are the main risk factors? Goal: to increase information and awareness. The group is divided in 3 subgroups. Each group writes down the risk factor of 1 of the 3 diseases they know on a paper. The physician or nurse discusses the results. A quiz about the main risk factors; smoking, lack of exercise, and diet (salt, fat, high energy). Correct answers and some extra information are provided by video.
4. 6-min walking test. Material: numbers, a flat track of 50 m marked by a line at the start and turning point. Marks on every 10 m on the track. Chronometer, whistle. Instruction: walk, not run, the maximum distance in 6 min. Give a time indication every minute: still 5 min left … , 4 min left … , 3 min left …. , 2 min left … , 1 min left … STOP. Participants stop and remain at their place. Moderator counts each passages at the starting point, each time 100 m, and add the distance of the last round. Goal: to provide a quick, approximate measurement of physical fitness [20–21].
5. What are my personal risk factors? Method: place 4 large pieces of paper at each corner of the room: (i) lack of physical exercise, (ii) smoking, (iii) high dietary salt, and (iv) overweight. Participants put their name on each piece of paper, as appropriate.
6. Personal plan for change. What is the risk factor I will work on? What action will I take (when, how, where)? Do I need support to reach my goal from the CHSC or community? Write the details on paper, 1 to keep yourself and 1 for the administration of the CHSC.
7. Each participant tells the group in one 1 min about her/his plan. Moderator asks additional questions to make plan more concrete.
8. End. Fill in database. (Date, 6-min walking test and lifestyle intervention of choice.)

### Implementation and Evaluation of *Dutch*-*China CPP*

Following the piloting and further adaptation, the program was implemented in a further seven communities in three cities from October 2012 to March 2013. Once agreement and commitment were obtained from each of the city health bureaus and CDCs, the staff from the relevant CHSCs received a 2-day training program. The overall goal was to evaluate the program reach, implementation, acceptability, and feasibility of program delivery by the CHSCs in each of the seven communities. Individuals were eligible for involvement in the program if they were more than 35 years old and if they lived in a neighborhood served by one of the participating CHSCs. Using the general guidelines provided by the team, each CHSC then developed its own tailored strategies for recruiting participants from their community in order to achieve the following three targets, that is, at least: (1) 1000 collected questionnaires, (2) 100 consultations in “high-risk individuals,” and (3) 30 “high-risk individuals” attending a lifestyle class. Participant recruitment was undertaken by community health workers. The general guidelines for recruitment were (1) identification from residents health records in CHSC and interviewed by phone; (2) face-to-face interview during routine home visits for other primary care purposes; (3) face to face recruitment during outpatient clinic attendance; and (4) distribution and collection of questionnaires in public community settings, such as community square, shopping center and free market. The process took between 2 to 4 weeks in each of the community health service centers.

### Evaluation of Whether Program Targets Were Achieved

Each CHSC was requested to enter the data for each participant’s questionnaires, consultations, and lifestyle classes into a Web-based software package. De-identified data were extracted from this database for statistical purposes. In addition to describing the overall study sample as well as details for all of the participants from each CHSC, these data were also used to calculate how well each CHSC performed in relation to the three targets. These were also used to calculate a test–re-test reliability check of the questionnaire score between steps 1 and 2.

### Surveys of Health Professionals and Participants

Additional surveys were conducted with health professionals from the CHSCs and program participants in order to evaluate feedback on their perceptions of the acceptability and feasibility of the program. The staffs from the participating CHSCs were asked a series of closed questions regarding the training, the implementation of each of the three stages of the program, the use of the Web-based database, as well as an overall evaluation of the program. They were also asked to indicate at least one positive and negative aspect of the program as open question. To assess the acceptability of program by participants, a survey was conducted among some participants with a risk score of at least 5 at the end of the program. Additionally, a year after the completion of the program was completed, provincial CDC staff conducted face to face interviews with program participants in each of the seven communities. Three subgroups were surveyed: (1) those participants who only completed a questionnaire (step 1), (2) those who also attended a follow-up consultation (steps 1 and 2) but no lifestyle class, and (3) those with participants who attended all three steps (steps 1–3). Three hundred participants were randomly chosen from the program database to represent these three groups from the seven communities. Of the 300 selected for interview, one third of these (*n* = 100) could be contacted and agreed to answer a set of multiple choice questions. These individuals were asked their opinions about the purpose of the program, the reasons why they did/did not attend consultations and/or lifestyle classes, as well as some additional questions about changes in their lifestyle over the previous year.

### Statistical Analysis

For all statistical analyses, SPSS Statistics 20.0 for Windows was used: (1) For the analyses related to the SAQ’s, consultations and lifestyle classes statistical significance was tested by either a comparison of means or a comparison of proportions. (2) Because of the limited number of participating CHSC’s, no statistical analysis was performed in the survey of health professionals. (3) In the survey of participants, statistical significance was tested by the Chi-square test for trends.

## Results

### How Many People Participated at Each Stage of the Program?

**Fig. 2:**
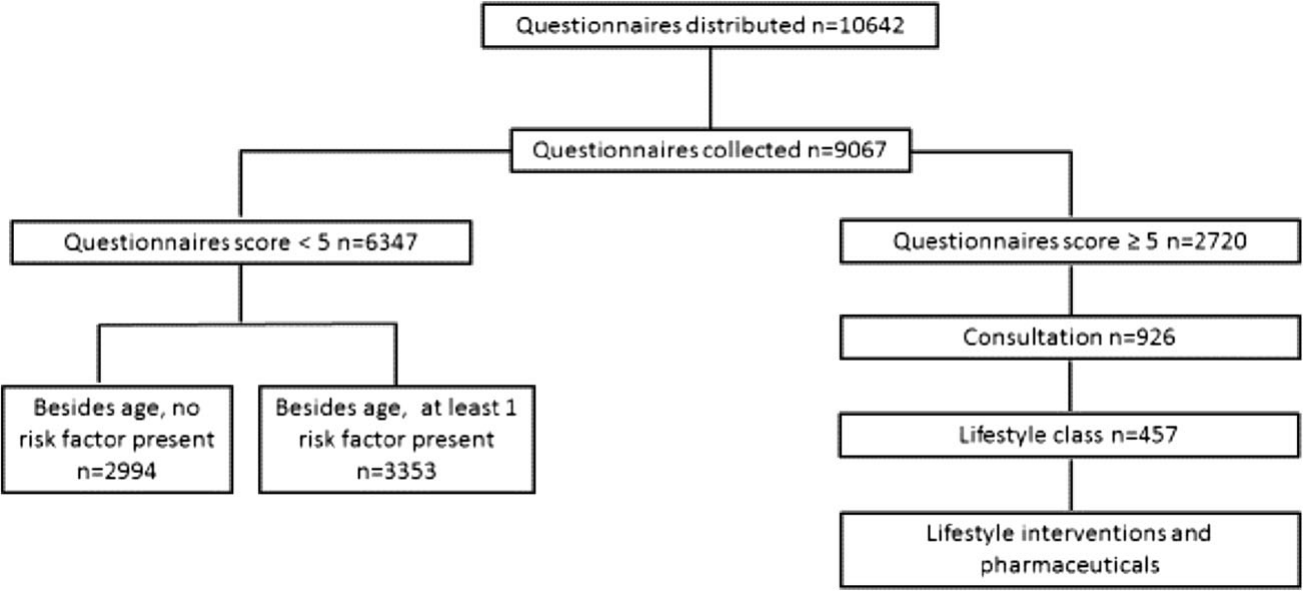
Summary flowchart of evaluation findings

Figure 2 summarizes the number of participants who underwent initial screening (*step 1*) and participated in each of the subsequent stages of CVD high-risk assessment (*step 2*) and lifestyle education and counseling (*step 3*). A total of 10,642 China questionnaires were distributed by CHSCs in seven communities, and 9067 completed questionnaires were collected. A total of 2720 participants (30 %) had a score equal to or above the threshold value of 5; CHSC staff invited all of these to attend a consultation and lifestyle education sessions. Of these, 926 participants (34 %) received a consultation and 457 of these individuals (44 %) also attended a lifestyle education session.

### Were the Program Targets Achieved for Each CHSC?

Table 3 summarizes the number of participants for each CHSC at the different program steps. The table demonstrates that five of the seven CHCs easily reached the three designated targets; one CHC was very close to achieving these and one did not achieve any of the targets. This latter CHSC excluded all participants who already had a personal history, while CHSC 6 emphasized face-to-face interviews with prospective participants followed by an immediate consultation, if that was appropriate. Compared with the other study communities, CHSC 6 is also in a more rural community where most of the doctors from the Community Health Service Center also live in the same community and have a very good interaction with the local citizens.

**Table 3:** Program targets for each of the seven CHSCs

Items	Questionnaires delivered (*N*)	Questionnaires returned (*N*)	Consultations performed (*N*)	Lifestyle classes attended (*N*)	Number of targets reached (*N*)
		Target = 1000	Target = 100	Target = 30	Target = 3
CHSC 1	1500	1244	189	32	3
CHSC 2	1560	1554	205	40	3
CHSC 3	2100	2034	101	45	3
CHC 4	1200	1029	62	66	2
CHSC 5	1233	996	33	25	0
CHSC 6	1049	1047	222	212	3
CHSC 7	2000	1163	114	37	3
Total	10642	9067	926	457	5

### Who Were the Program Participants?

The characteristics of program participants are summarized in Table 4. Men and women were almost equally represented (52 vs. 48 %, respectively) and had a similar age distribution. Participants’ mean age was 52.4 years, 40.3 % were over 55 years old; 33.5 % of the participants presented overweight and 6.9 % obesity and 24.4 % reported smoking.

**Table 4:** Characteristics and risk factors of screened participants

Items	Total *N* (%)	Female *N* (%)	Male *N* (%)
	9067(100)	4685 (51.7)	4382 (48.3)
Age			
35–44	2903 (32)	1462 (32.4)	1441 (33.4)
45–54	2509 (27.7)	1267 (27.0)	1242 (28.3)
≥55	3579 (40.3)	1902 (40.6)	1677 (38.3)
Mean age (95 %) CI)	52.4 (52.1–52.7)	52.6 (52.2–53.0)	52.3 (51.9–52.7)
BMI			
<24	5404 (59.6)	2882 (61.5)	2522 (57.5)
≥24 and <28	3041 (33.5)	1455 (31.1)	1586 (36.2)
≥28	622 (6.9)	348 (7.4)	274 (6.3)
Mean BMI (95 % CI)	23.5 (23.4–23.6)	23.4 (23.3–23.5)	23.6 (23.5–23.7)
Smoking**	2212 (24.4)	136 (2.9)	2076 (47.4)
Family history	1848 (20.4)	917 (19.6)	931 (21.2)
Personal history	1766 (19.5)	881 (18.8)	885 (20.2)
Risk score ≥5**	2720(30)	1025(21.9)	1695 (38.7)

Mean age, BMI, and scores: significance by comparison of two means. Rate of smoking, personal, and family history, scores ≥5: significance by comparison of two proportions

**p < 0.001

Chi-square test with exact probability to test differences of rates of smoking and personal and family history, and one-way analysis of variance were performed to compare the differences of means of age, BMI, and scores between male and female, respectively. Compared with women, men had a significantly higher smoking rate (47.4 vs. 2.9 %), and this risk factor is largely responsible for the higher proportion of males having a risk score of ≥5 (38.7 vs. 21.9 %); 20.4 % participants had a family history of hypertension, diabetes, ischemic heart disease, and/or stroke; and 19.5 % had a personal history, with no gender difference.

In the group with a risk score of at least 5, the majority (61.2 %) already had a personal history because each CHSC used the recruitment guidelines to develop their preferred recruitment strategies to achieve the required target. Some of the CHSCs chose to include those individuals who already had a personal history. In Table 5, risk factors are shown in groups with a low- and high-risk scores combined with or without a personal history of hypertension and/or diabetes. For those with no personal history but a high-risk score, all risk factors are more prevalent, compared with the group with a low-risk score. Besides smoking rate, all the other risk factors are quite similar in the high-risk group with or without a personal history. In any case, participants who already have a personal history can still reduce their future risk of disease by making appropriate lifestyle changes.

**Table 5:** Difference in risk factors between the groups with a score <5 and a score ≥5

Items	No personal history and a score <5 (*n* = 6245)	No personal history and a score ≥5 (*n* = 1056)	Personal history and a score <5 (*n* = 102)	Personal history and a score ≥5 (*n* = 1664)
Smoking in %	14.7	81.0	0.0	26.5
Family history in %	13.9	36.2	8.8	35.3
Mean age in years	49.1	57.3	45.5	62.2
Mean BMI in units	22.9	24.8	23.1	24.8

Note: All differences are significant at *p* < 0.001. Smoking rate and family history: significance by comparison of two proportions. Mean age and BMI: significance by comparison of two means

### Characteristics of High-Risk Population Attending a Consultation

Nine hundred twenty-six participants whose SAQ score was ≥5 attended preventive consultations in CHSCs. More than half (52.6 %) had a history of hypertension and 13.5 % diabetes. Almost one quarter of those with a history of hypertension still had uncontrolled hypertension and about 10 % of diabetes patients still had a high glucose level. However, among the assessed individuals, we only identified 11 additional individuals with hypertension, 25 with diabetes, and 32 with pre-diabetes.

### Uptake of Lifestyle Education Sessions

More than 450 (*n* = 457) participants whose risk scores were ≥5 attended lifestyle classes led by CHSC professionals. According to the personal action plans made by individuals, the CHSCs provided further lifestyle intervention programs related to healthy cooking and diet, increasing physical activity and quitting smoking. The majority of males (65 %) chose the quit smoking intervention group and most women (54 %) chose the exercise group.

### Reliability of the Self-Administered Questionnaire

During the face-to-face consultation, the items of the questionnaires were repeated, in addition to the clinical measurements, therefore making it possible to assess the test–re-test reliability of the completed. In the majority of individuals (93 %), where individuals scored 5 or higher and returned for a consultation, they were re-confirmed as being high risk (Table 6).

**Table 6:** Relationship between self-assessment of risk factors and re-assessment by a physician

Items	SAQ screening *N* (%)	Re-check SAQ and clinical measurement by physicians *N* (%)	Consistency rate (%)	95 % confidence interval
Smoking	2212 (24.4)	2132 (23.5)	96.4	95.2–97.6
Family history	1850 (20.4)	1724 (19.0)	93.2	91.6–94.8
Personal history	1786 (19.5)	1659 (18.3)	92.9	91.0–94.8
BMI >24	3663 (40.4%)	3238 (35.7)	88.4	86.3–90.5
Correct classification as high risk			93.0	91.4–94.6

### Survey of CHSC Staff

Representative health professionals from all the seven CHSCs returned questionnaires asking them about their feedback on the program. Firstly, they were asked to give an opinion on seven statements. The implementation of each of the steps of the program, the support by the project management, and the Web-based database all received positive feedback from those interviewed. However, the majority of the CHSC staff agreed that the financial remuneration was insufficient to cover the extra work and costs. Secondly, community health staffs were also asked to identify the most positive and negative aspect of the program. The positive aspects included the following: *1*) *This is a good approach for detecting participants*’ *risk*; *2*) *The approach used was simple*, *convenient, and practical to use*; *3*) *It was an effective approach for improving residents*’ *unhealthy lifestyle and raising their awareness*; *4*) *The lifestyle education sessions and intervention activities were helpful in improving lifestyle*; *and 5*) *This program approach is very innovative in China*. The negative aspects included the following: *1*) *The funding to cover expenses and our efforts is insufficient*; *2*) *Follow*-*up of participants during the different stages of the intervention is difficult*; *3*) *It can be difficult to generate awareness*; and *4*) *There is now a need for scaling up*.

In summary, while the respondents from the CHSCs were pleased with the design and concept, most felt strongly that the remuneration and program implementation required some refinements and improvement.

### Survey of Program Participants

The survey of program participants indicates that 71 % of the respondents agree about the necessity of this intervention, 13 % take a neutral position, and 16 % have a negative opinion. The two most frequent reasons for not attending a consultation and/or lifestyle class are the time and place was not convenient (about 70 %) and a substantial part (about 20 %) stated they do not have a problem and by consequence there is no need for further interventions. Only a few participants, less than 10 %, stated they did not receive an invitation. The reasons for attending a consultation and/or lifestyle class are the expectation of positive impact on their health (about 80 %), the personal invitation by the local CHSC staff (about 60 %), and the convenient time and place of the interventions (about 40 %).

Table 7 summarizes self-reported changes in lifestyle in the past year. Chi-square statistic is performed to test trends of behavior change in different groups. As we predicted that consultations (step 2) and lifestyle classes (step 3) might have more impact on those who participated, we allocated a value of zero to respondents who only returned a questionnaire, a value of 1 for those attending a consultation and a value of 2 for those who also attended a lifestyle class. For smoking, we performed the statistic test based on the total number minus the respondents who never smoked and we tested for smoking cessation and a combined outcome of smoking cessation and a decrease in the number of cigarettes. Also for alcohol use, the statistical test is based on the number of alcohol users. Although most of the tests for each specific lifestyle change are not statistically significant, the combined outcome of at least one lifestyle change is highly significant and also the reported influence of the program is more substantial, if participants attended steps 2 and 3.

**Table 7:** Self-reported lifestyle change of participants in different program stages

Items	Group 1 (only step 1; *N* (%))	Group 2 (steps 1 and 2; *N* (%))	Group 3 (steps 1–3; *N* (%))	*p* value (Chi-square for trends)
Number (all respondents)	48	26	26	
Physical exercise: increase	11 (23 %)	8 (31 %)	14 (54 %)	0.03 (7.38)
Less dietary salt	26 (54 %)	14 (54 %)	19 (73 %)	0.24 (2.88)
Weight loss	13 (27 %)	7 (27 %)	9 (35 %)	0.76 (0.54)
Number (never smoked excluded)	24	10	18	
Smoking cessation	3 (13 %)	2 (20 %)	5 (28 %)	0.46 (1.55)
Smoking: decrease	10 (42 %)	7 (70 %)	11 (61 %)	0.24 (2.86)
Number (no alcohol excluded)	22	14	12	
Alcohol: decrease	10 (45 %)	7 (50 %)	10 (83 %)	0.09 (4.84)
Number of respondents	48	26	26	
Lifestyle change (at least 1)	31 (65 %)	22 (85 %)	25 (96 %)	0.01 (10.69)
Lifestyle change by the program	18 (38 %)	15 (58 %)	21 (80 %)	0.01 (12.90)

## Discussion

China has experienced a rapid increase in chronic NCDs such as cardiovascular diseases and diabetes over the past 20 years. The effects of the epidemic of chronic NCDs on the healthcare system and the economy are already very significant [6, 7]; hence, there is an urgent need to adapt, refine, and scale-up approaches to prevention that have been developed and well evaluated in Western countries over the past 40 years. One approach is to use community-based approaches that can effectively reduce cardiovascular disease and related cardio-metabolic risks in high-risk individuals in ways that are consistent with the changing healthcare system in China. However, the evidence concerning these kinds of approaches in China is still quite limited [22, 23]. The aim of the China-Dutch cooperation program has been to demonstrate the feasibility and acceptability of culturally adapting a Dutch approach called, *Prevention*-*Consultation* [13, 17], which is based on an approach to prevent cardiovascular disease, diabetes and other related conditions that has been developed and very well evaluated in high income countries over more than 20 years. The approach that has been adapted for this program involves three steps, (1) self-assessment of CVD risk, (2) follow-up consultation to confirm risk, and (3) lifestyle education and referral for further intervention activities being undertaken in the community. It is important to emphasize that the aim of this evaluation was to focus primarily on evaluation of the reach, uptake, and adoption of the different program components under very “real-world” conditions.

In summary, the evaluation of program implementation indicates that this real-world program has quite good reach, uptake, and adoption of the different program elements. Five out of seven CHCs reached all of the targets that were set for reach, adoption, and uptake, and one missed by only one target. The remaining CHC demonstrated poor implementation; this occurred as a result of a misunderstanding because this CHSC excluded participants who already had a personal history of hypertension and/or diabetes. Consequently, only 10.5 % of the respondents from this CHSC reached the risk score threshold of 5. Overall though, the screening questionnaire was completed by 9067 participants in seven demonstration communities and 30 % of these individuals were defined as high risk according to their scores. All of these high-risk individuals were invited to undergo further clinical and laboratory tests and one third took up this invitation. Almost half of those who were re-screened, participated in lifestyle education classes, that is, almost one fifth of those who were initially scored as high risk. Although the final proportion of individuals attending consultations and lifestyle classes is less than ideal, this is still very comparable with the experience of western countries [17, 24–26] and indicates great potential for scale up in the future. However, as the awareness of NCDs increases across all sections of society and there is a much more integrated and coordinated approach to prevention programs in the coming years in China, the authors predict that the uptake, engagement, and involvement in such programs will also improve a lot in the future.

Despite the high proportion of people who already had a personal history of hypertension and diabetes, participants with a score of 5 or more still showed an overall high-risk profile with smoking in males and overweight in both sexes being two very important risk factors related to NCDs in China [6, 7, 9, 10]. An important finding from our study was that the Cardiovascular Disease Risk Management tool (CVDRM-SAQ), adapted from the Netherlands [13, 17], was easy to use tool and it also demonstrated reasonable test–re-test reliability. A majority of cases (93 %), where individuals scored 5 or higher and returned for a consultation, were also confirmed as being high risk. In China, a national cohort study of the development of a population-based CVD high-risk screening tool has been conducted over 15 years [15, 16]. This tool includes seven items (age, sex, BP, serum total cholesterol, BMI, smoking, and having diabetes); however, to use this tool, it requires all individuals to have clinical and laboratory tests undertaken by a physician. Our adapted CVDRM-SAQ is similar to this tool, but a two-step risk evaluation process is used. With only those individuals with a high-risk score being referred for further tests, this significantly limits the workload and costs. However, of course, some participants will underestimate and/or under-report their level of risk and consequently, are not referred for further tests and counseling. Of course, this approach should be further validated in China in the future in order to properly evaluate the sensitivity and specificity of the two-step risk stratification approach that has been evaluated for its acceptability and feasibility in this real-world implementation trial.

While the feedback from CHSC staff was quite positive, some did raise concerns about funding issues and long-term program sustainability. Their responses are indicative of this being a new model and approach in China; hence, the uncertainty about how this might become a more routine activity. Clearly, recalling patients for further consultations and lifestyle classes was difficult and time consuming. However, all except one of the CHSCs understood the clear rationale for this new approach in China. This trial suggests the already existing institutions, a network of CHSCs steered by the CDC structure, are capable to fulfill these new tasks if the some critical conditions, like sufficient financial support, can be addressed.

Moreover, the recent policies of China’s National Health Reform encourage the primary care CHSCs to serve as “health gatekeepers” and to provide primary care for community residents [27]. This includes the government providing ¥35/person (approximately, US$ 5.5) to CHSCs to deliver a defined package of 11 basic public health services that include NCD prevention and management in terms of yearly clinical assessment, monthly blood pressure monitoring, quarterly blood sugar assessment, health education addressing healthy diet, physical activity, and medication adherence, and routine home follow-up visits. If this new approach would be combined with clinical follow-ups and lifestyle interventions, CHSCs could be able to meet the goals set by the Chinese government authorities.

The feedback from program participants was also quite positive; however, it should be acknowledged that this feedback was self-reported and not independently verified. Participants were generally very positive about the approach that was used, and those who participated in steps 2 and 3, more frequently reported positive changes in their lifestyle. Although the short term positive trends look promising, it is important that future research should properly evaluate the longer term behavioral and clinical outcomes of this approach.

There were also some of other important system-level factors contributing to the success of the program. These included the strong commitment and support of local government and the different levels of the CDC. However, it was also important that the program was so carefully adapted and further developed during the 2-year piloting phase before the formal implementation trial commenced. This phase also involved a lot of interaction and collaboration with each of the CHSCs and their staff. Each of these steps further ensured that the program was not only appropriate for the local context but would also increase the likelihood that this program will be more scalable in the future.

### Limitations and Future Challenges

The authors acknowledge that there are a number of limitations and challenges which require further research and investigation. Firstly, it is important to undertake more controlled implementation research in order to improve the program and to evaluate the extent to this which program impacts on lifestyle behaviors and cardio-metabolic risk in both the short and longer term. It is also important to conduct more research to validate further the self-assessment tool. Secondly, it is important to identify strategies and approaches that might boost the number of individuals who might attend for a more detailed behavioral and clinical assessment after they have self-completed the checklist and they have been provisionally identified as being high risk. Thirdly, it is also important to undertake more research on how to boost the uptake of lifestyle change and its long term maintenance. For example, there are other more contemporary and acceptable ways of providing the education and counseling by the use of information and communications technology and the internet. Of course, these issues and challenges are not unique to China, and indeed, they have been identified in relation to similar programs in other countries [17, 24–26]. Finally, as the broader national health reforms continue to be rolled out in China in order to improve the management and control of chronic conditions like diabetes and cardiovascular disease, it will also be important to develop strong linkages between this kind approach to chronic disease prevention with education and strategies that are focused on both primary prevention as well as efforts to improve the ongoing management and treatment of chronic conditions like diabetes.

## Conclusions

China and other developing countries are facing an epidemiological transition resulting in a rapid increase in NCDs. Consequently, there is an urgent need for cost-effective and scalable interventions to meet the goals set by the WHO [5]. Our real-world evaluation of the implementation of a cardio-metabolic screening and intervention program adapted from the Netherlands and implemented in the seven communities in the Anhui province, China provides some initial positive findings regarding it feasibility and acceptability to program participants and health professionals. Although more research and evaluation of this approach is required, this approach might offer a valuable alternative strategy to reduce the risk of cardio-metabolic and other chronic conditions in rapidly developing countries like China in the future.
